# Sucrose synthase cleavage dominance underpins high-sugar phenotype in pitaya (*Hylocereus* spp.): a multi-genotype study integrating morphology, spatial sugar profiling, and enzyme activities

**DOI:** 10.3389/fpls.2026.1759042

**Published:** 2026-05-07

**Authors:** Renzhi Huang, Dandan Wu, Fanjin Peng, Guangzhao Xu, Zhuanying Yang, Xiaolong He

**Affiliations:** 1 College of Coastal Agricultural Sciences, Guangdong Ocean University, Zhanjiang, China; 2 Guangdong Engineering Technology Research Center of Tropical Crops High-Efficient Production, Zhanjiang, China; 3 South Subtropical Crops Research Institute, Chinese Academy of Tropical Agricultural Sciences, Zhanjiang, China

**Keywords:** enzyme activity, fruit quality, genotypic variation, pitaya, principal component analysis, sucrose metabolism

## Abstract

**Introduction:**

Pitaya (*Hylocereus* spp.), a rapidly expanding tropical fruit crop, faces a critical industry challenge: high yields often come at the expense of sweetness, limiting consumer appeal and market value. This study tests the hypothesis that cultivars with contrasting sugar phenotypes exhibit distinct profiles of sucrose-metabolizing enzyme activities, and that the balance between sucrose synthase (SS) cleavage and synthesis directions determines final sugar accumulation and spatial distribution patterns.

**Methods:**

We compared four phenotypically diverse cultivars selected based on preliminary field screening of three key traits: flesh color (white vs. pigmented), average fruit weight (small, medium, large), and soluble solids content (low, medium, high)—'Jindu No.1', 'White Crystal', 'Bicolor Fruit', and 'Bai Yulong'—through comprehensive assessments of morphological traits, soluble sugar profiles, and activities of key sucrose-metabolizing enzymes (neutral invertase [NI], acid invertase [AI], sucrose synthase [SS] in synthesis and cleavage directions, and sucrose phosphate synthase [SPS]) .

**Results:**

One-way ANOVA revealed significant genotypic variation in all measured traits, with sugar accumulation peaking in central fruit sectors and correlating strongly with enzyme activities. Notably, high SS-cleavage activity in 'Jindu No.1' and 'Bai Yulong' facilitated hexose buildup, while elevated SS-synthesis in 'Bicolor Fruit' diverted carbons away from sugars. Principal component analysis (PCA) integrated these traits, ranking quality as 'Bai Yulong' > 'Jindu No.1' > 'White Crystal' > 'Bicolor Fruit' and defining a "high-sugar" metabolic mode characterized by dominant SS cleavage.

**Discussion:**

These findings reveal physiological mechanisms driving quality differences and provide actionable biomarkers for molecular breeding of sweeter pitaya varieties, advancing sustainable industry development.

## 1 Introduction

Pitaya, also known as dragon fruit (*Hylocereus* spp.), has emerged as a high-value tropical fruit with global cultivation expanding rapidly due to its nutritional benefits and market demand (Ibrahim et al., 2018). Originating from Central America, major production hubs in China include southern provinces like Guangdong and Hainan, where annual output has surged in recent years (Wen and Liu, 2024). However, as the industry scales up, a pervasive issue has arisen: many varieties exhibit ‘large but not sweet’ traits, where increased fruit size compromises flavor quality, particularly sweetness, thereby constraining consumer satisfaction and premium pricing. This phenomenon reflects a fundamental trade-off in artificial selection: breeders have historically prioritized yield and fruit size, often at the expense of flavor-related traits such as sugar content (Tieman et al., 2017; Klee, 2010). In tomato, for example, selection for larger fruits has been associated with reduced sugar concentrations due to dilution effects and altered source-sink relationships (Schouten et al., 2016). Pitaya, as a recently domesticated and rapidly expanding crop, provides an ideal system to investigate whether similar trade-offs operate and whether metabolic adjustments—particularly in sucrose partitioning—can reconcile size and sweetness. This ‘big-but-not-sweet’ bottleneck underscores the urgent need for research to enhance intrinsic quality without sacrificing yield.

Fruit quality in pitaya is predominantly determined by soluble sugars—sucrose, glucose, and fructose—which not only dictate sweetness but also influence texture and nutritional value (Wichienchot et al., 2010). Sugar accumulation is not a passive process but an actively regulated metabolic pathway involving key enzymes: neutral invertase (NI) and acid invertase (AI) hydrolyze sucrose into hexoses; sucrose synthase (SS) operates bidirectionally for synthesis or cleavage; and sucrose phosphate synthase (SPS) drives sucrose formation (Sturm, 1999; Stein and Granot, 2019). Studies in other fruits, such as citrus and apple, have shown that enzyme activities directly correlate with sugar profiles, highlighting genotypic regulation as a key driver of quality variation (Lowell et al., 1989; Huber and Huber, 1996). The mechanistic basis for genotypic variation in sugar accumulation lies in the differential expression and regulation of sucrose-metabolizing enzymes. Sucrose synthase (SS), in particular, exhibits bidirectional activity that can shift carbon flux toward either hexose production (cleavage direction) or sucrose synthesis and structural carbon allocation (synthesis direction). In many fruit crops, including tomato, citrus, and apple, allelic variation in SS genes or differential isoform expression has been shown to alter sink strength and final sugar composition (Stein and Granot, 2019; Ruan, 2014). Similarly, invertases (NI and AI) and sucrose phosphate synthase (SPS) are subject to developmental and environmental regulation, with genotypic differences in their activity profiles directly impacting hexose accumulation. Therefore, we hypothesized that pitaya cultivars with contrasting sugar phenotypes would exhibit distinct patterns of enzyme activity, particularly in the balance between SS cleavage and synthesis, and that these patterns would correlate with spatial sugar distribution and overall fruit quality.

Despite advances in pitaya research, including germplasm diversity (Pan et al., 2017) and nutritional profiling (Nishikito et al., 2023), gaps persist. Existing studies often focus on single-variety sugar dynamics or nutritional components, lacking systematic comparisons of sucrose metabolism across genotypes (Nomura et al., 2005; Zhang et al., 2022). No prior work has integrated morphological traits, sugar distribution, and enzyme activities to elucidate the physiological basis of quality differences, impeding targeted breeding.

To address these gaps, we selected four cultivars with contrasting phenotypes: ‘Jindu No.1’ (red-fleshed, high sweetness but smaller size), ‘White Crystal’ (white-fleshed, moderate sugar and size), ‘Bicolor Fruit’ (pigmented flesh, lower sugar potential), and ‘Bai Yulong’ (white-fleshed, large size with balanced quality). These four cultivars were deliberately chosen to represent the major phenotypic categories in commercial pitaya production: high-sugar but small-fruited (Jindu No.1), large-fruited with balanced quality (Bai Yulong), moderate traits (White Crystal), and pigmented but low-sugar (Bicolor Fruit). This selection enables a comparative analysis of how divergent morphological and metabolic traits relate to sucrose metabolism, thereby testing our central hypothesis (Esquivel et al., 2007). Our objectives were twofold: (i) to quantify genotypic differences in morphology, sugar components, and enzyme activities; and (ii) to uncover metabolic mechanisms, particularly SS functional divergence, informing a “high-sugar” mode for breeding superior varieties.

## 2 Materials and methods

### 2.1 Plant materials

**Figure 1:**
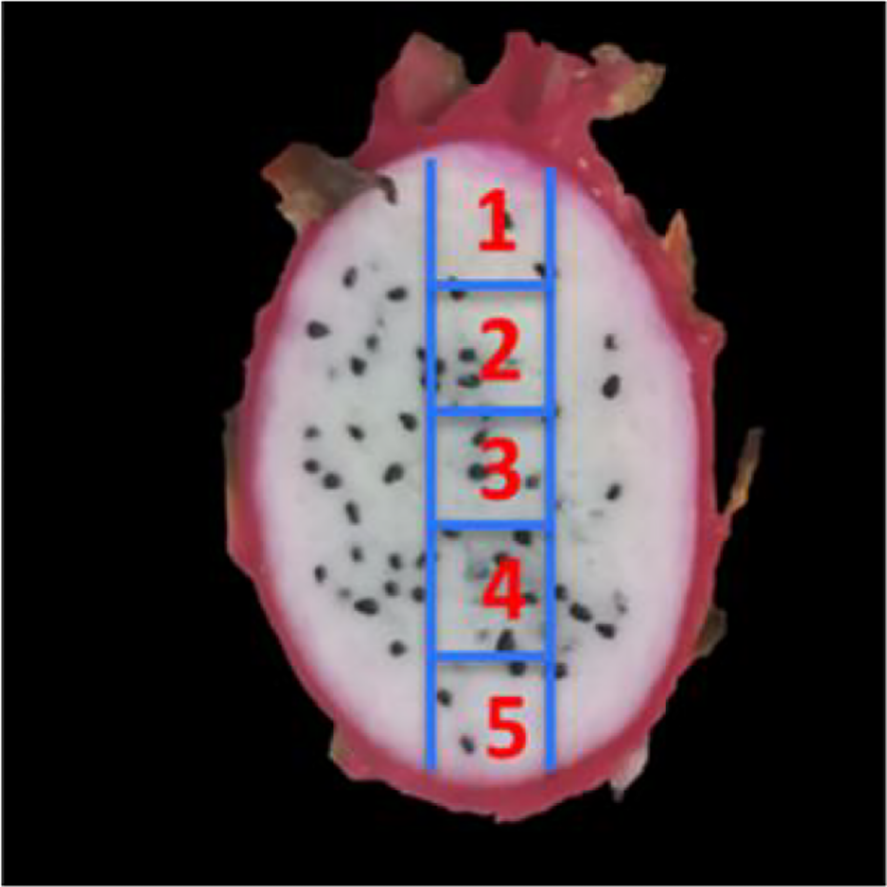
Schematic diagram of sample site division. Sector 1-5.

To ensure genotypic differences were not confounded by environmental factors, fruits were sourced from a uniform cultivation site at Zhanjiang Yongheng Agricultural Technology Co., Ltd. (Zhanjiang, China), under identical management practices (e.g., irrigation, fertilization). The four cultivars—’Jindu No.1’, ‘White Crystal’, ‘Bicolor Fruit’, and ‘Bai Yulong’—were selected based on preliminary field evaluations of flesh color, fruit weight, and soluble solids content (Brix) conducted over two growing seasons. These cultivars represent the major phenotypic classes in pitaya germplasm: high-sugar with moderate size (‘Jindu No.1’), large fruit with balanced quality (‘Bai Yulong’), intermediate traits (‘White Crystal’), and pigmented flesh with low sugar potential (‘Bicolor Fruit’). This deliberate selection allowed us to test the hypothesis that genotypic differences in sucrose metabolism underpin sugar accumulation patterns. Fruits were harvested at full maturity (approximately 30–35 days post-anthesis) from healthy plants. For each cultivar, five fruits were collected as biological replicates for morphological measurements, and three fruits were randomly selected as biological replicates for sugar and enzyme activity assays. All fruits were washed, air-dried, peeled, and longitudinally sectioned into five equal parts (sectors 1–5 from top to base, as illustrated in **Figure 1**) for spatial analysis. Samples were flash-frozen at –40 °C until analysis.

### 2.2 Morphological measurements

**Figure 2:**
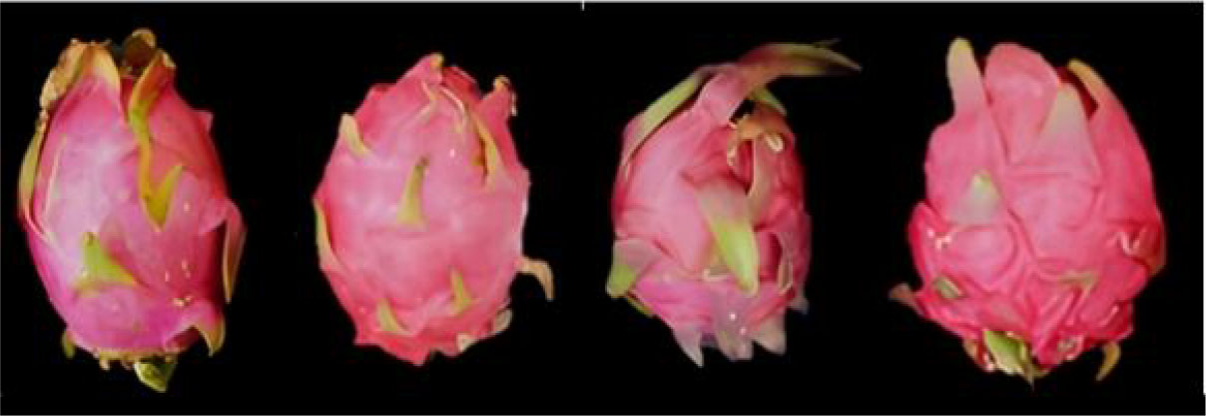
Morphological images of four varieties of pitaya (from left to right, they are ‘Bai Yulong’, ‘Bicolor Fruit’, ‘Jindu No.1’, and ‘White Crystal’).

To assess commercial quality and sink strength, morphological traits were measured using 5–10 fruits per cultivar (**Figure 2**). Fruit weight and peel weight were determined with a precision electronic balance (Model ME4002, Mettler Toledo, Switzerland; accuracy 0.01 g). Longitudinal and transverse diameters, along with peel thickness, were measured using a Mitutoyo digital caliper (0–150 mm, accuracy 0.01 mm). Edible rate (%) and fruit shape index were calculated as: Edible rate = [(Average fruit weight – Average peel weight)/Average fruit weight] × 100; Fruit shape index = Longitudinal diameter/Transverse diameter.

### 2.3 Soluble solids content

To evaluate overall sweetness potential, soluble solids were measured using an ATAGO handheld refractometer (Model PAL-1, ATAGO Co., Ltd., Japan; range 0–93% Brix). After zero calibration with distilled water, two drops of juice from each fruit sector were analyzed, with three replicates per sample.

### 2.4 Sugar components and enzyme activities

To investigate metabolic drivers of sugar accumulation, glucose and fructose contents were quantified, and activities of NI, AI, SS (synthesis and cleavage directions), and SPS were assayed following a modified protocol (Yang et al., 2013). The fruit was divided into five longitudinal sectors (top to base) to investigate spatial heterogeneity in sugar accumulation, as previous studies in pineapple and banana have shown that sugar content and enzyme activities can vary significantly along the proximal-distal axis due to differential vascular supply and sink activity (Zhang et al., 2012, 2022). Pulp samples (1 g) from each sector were homogenized in extraction buffer, and enzyme activities were expressed as mg·g^-^¹·h^-^¹ fresh weight. Each sample was measured in three technical replicates to ensure accuracy, and the mean values were used for statistical analysis.

### 2.5 Data analysis

Data were processed using Excel 2019 for summaries. Data normality was assessed using the Shapiro–Wilk test and all datasets met the assumption of normality (*p* > 0.05). Homogeneity of variances was confirmed using Levene’s test. One-way analysis of variance (ANOVA) followed by Tukey’s HSD *post-hoc* test was performed using GraphPad Prism 8.0.2 to compare means among cultivars and fruit sectors, with significance set at *p* < 0.05. F-values and degrees of freedom are reported for each analysis. Pearson correlation coefficients were calculated using SPSS 27.0 to assess relationships among traits, with significance levels indicated as *p* < 0.05 (*) and *p* < 0.01 (**). Principal component analysis (PCA) was performed on eight quality traits using SPSS 27.0. Data were standardized (z-scores) prior to analysis to ensure equal weighting of variables. Components with eigenvalues >1 were retained, and varimax rotation was applied to enhance interpretability.

## 3 Results

### 3.1 Morphological traits: the basis of phenotypic diversity

Significant genotypic variations were evident in morphological indicators (**Table 1**). Edible rates exceeded 70% across all cultivars. ‘Bai Yulong’ exhibited the highest fruit weight (237.32 ± 32.48 g), longitudinal diameter (106.97 ± 15.7 mm), and peel thickness (3.48 ± 1.89 mm), whereas ‘Jindu No.1’ had the thinnest peel (1.77 ± 0.61 mm). ‘Bicolor Fruit’ showed the lowest fruit weight (158.03 ± 28.47 g) but the highest fruit shape index (1.48 ± 0.12), indicating a more elongated morphology. These variations suggest a complex interplay between fruit morphology and internal quality, potentially influencing sink capacity for sugar accumulation. 

**Table 1:** Morphological indicators of four varieties of pitaya fruits.

Variety	Single fruit weight/g	Peel weight/g	Edible rate/%	Longitudinal diameter /mm	Transverse diameter /mm	Fruit shape index	Thick- skinned /mm
Jindu No.1	204.03 ± 69.91^a^	48.03 ± 16.72^b^	0.76 ± 0.22^a^	83.65 ± 24.6^b^	67.03 ± 19.83^a^	1.25 ± 0.37^a^	1.77 ± 0.61^a^
White Crystal	164.8 ± 21.95^a^	41.81 ± 6.02^b^	0.74 ± 0.04^a^	71.05 ± 4.01^c^	64.5 ± 3^a^	1.1 ± 0.04^a^	1.94 ± 0.47^a^
Bicolor Fruit	158.03 ± 28.47^a^	44.49 ± 8.13^b^	0.72 ± 0.04^a^	87.54 ± 7.87^b^	59.32 ± 3.09^a^	1.48 ± 0.12^b^	2.26 ± 0.66^a^
Bai Yulong	237.32 ± 32.48^a^	70.82 ± 20.14^a^	0.7 ± 0.11^a^	106.97 ± 15.7^a^	66.03 ± 6^a^	1.64 ± 0.34^b^	3.48 ± 1.89^a^

One-way ANOVA results: Fruit weight: F_3,16_ = 16.89, *p* = 0.001; Peel weight: F_3,16_ = 7.98, *p* = 0.002; Longitudinal diameter: F_3,16_ = 16.24, *p* = 0.001; Transverse diameter: F_3,16_ = 4.17, *p* = 0.023; Fruit shape index: F_3,16_ = 10.45, *p* = 0.001. Different lowercase letters indicate significant differences by Tukey’s HSD *post-hoc* test (*p* < 0.05).

### 3.2 Spatial heterogeneity of soluble solids and sugar components

**Figure 3:**
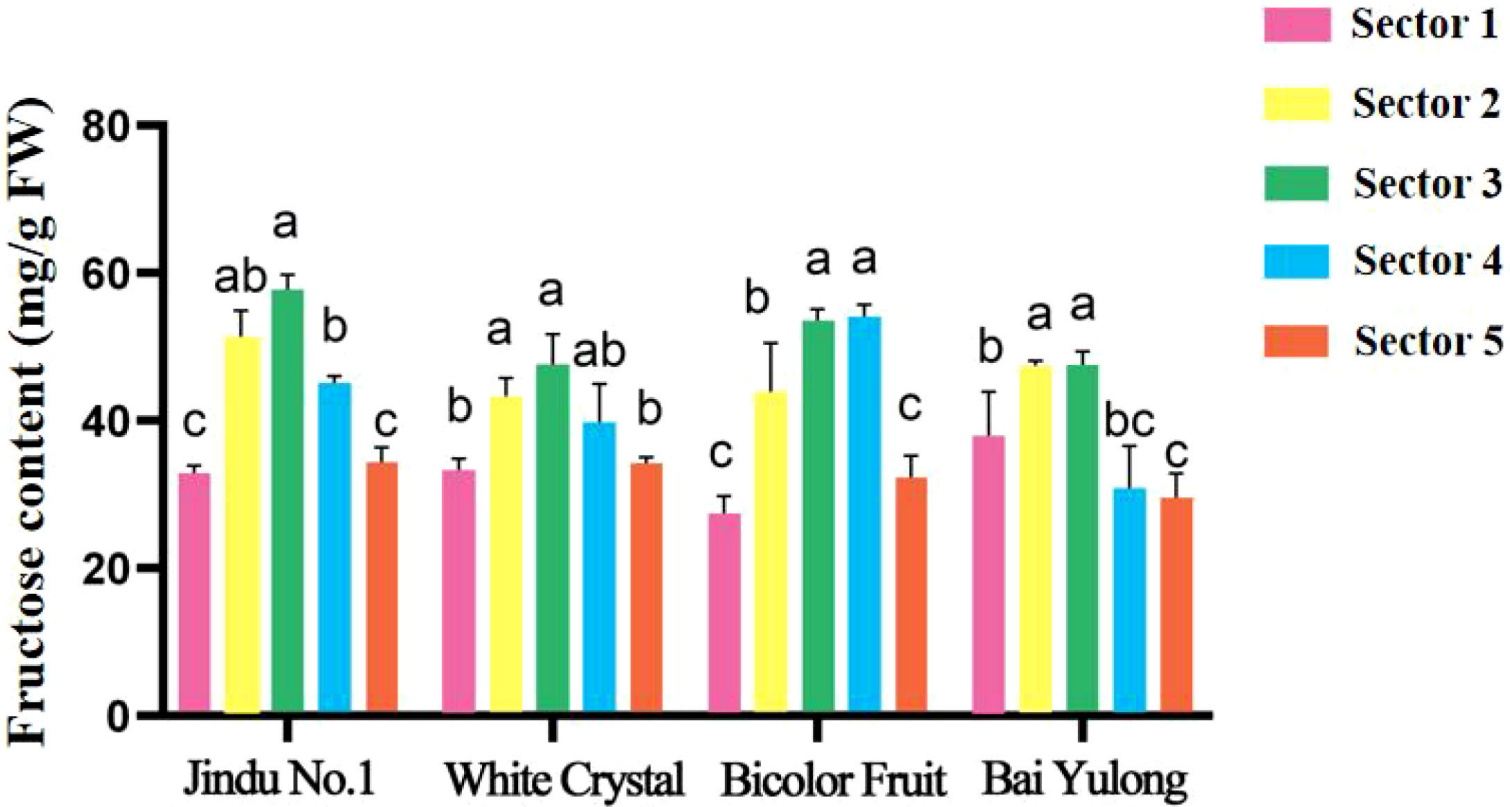
Fructose content in different parts of four varieties of pitaya fruits. Data are means ± SD (n=3). For each cultivar, one-way ANOVA was performed with fruit sector as the factor. Different lowercase letters indicate significant differences among sectors within the same cultivar (Tukey’s HSD, *p* < 0.05).

**Figure 4:**
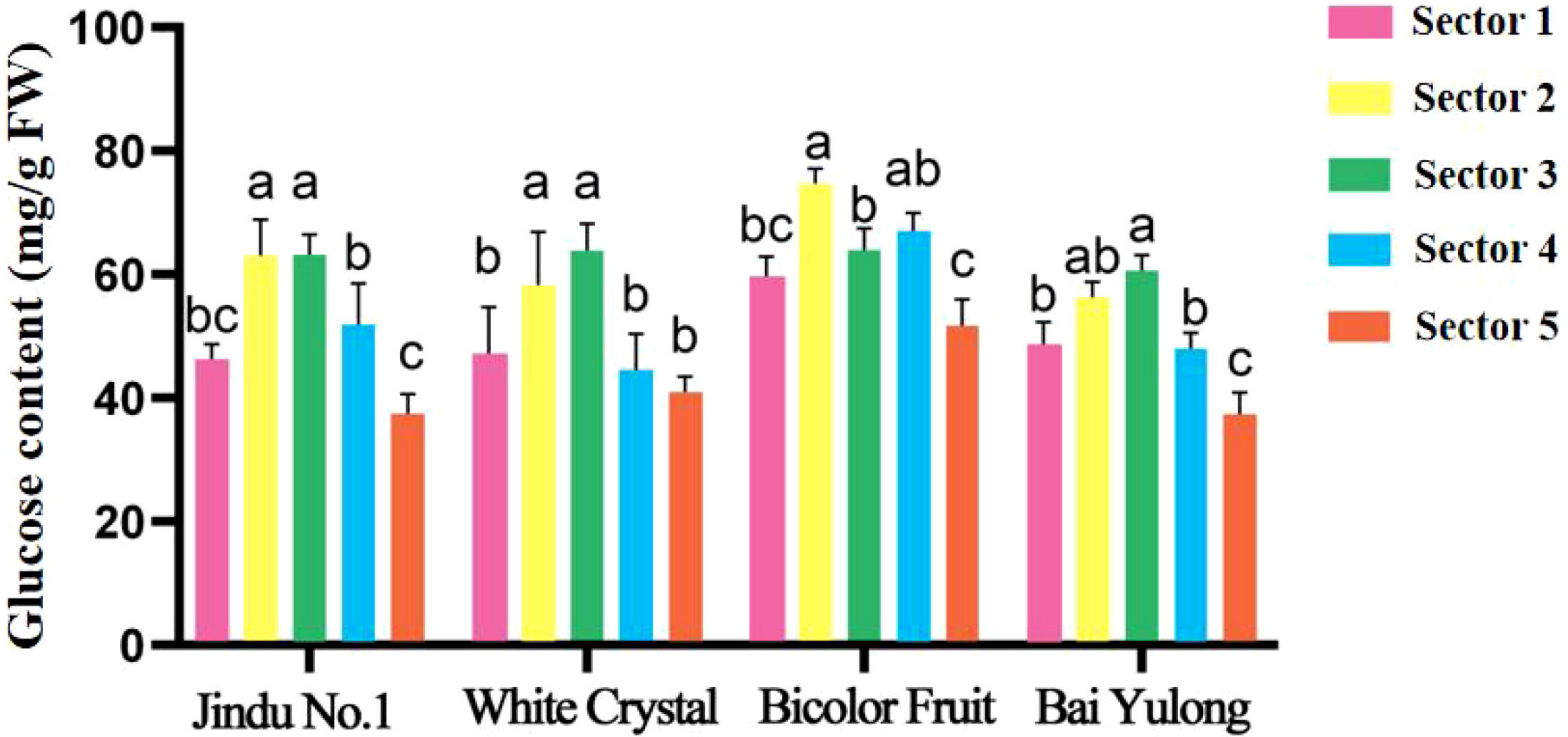
Glucose content in different parts of four varieties of pitaya fruits. data are means ± SD (n=3). For each cultivar, one-way ANOVA was performed with fruit sector as the factor. Different lowercase letters indicate significant differences among sectors within the same cultivar (Tukey’s HSD, *p* < 0.05).

To test for spatial heterogeneity within each cultivar and to compare cultivars at each sector, one-way ANOVA was performed with fruit sector or cultivar as the factor, respectively, followed by Tukey’s HSD *post-hoc* test (*p* < 0.05) for all analyses. Soluble solids content varied by cultivar and fruit sector, averaging 11.8–15.3% (**Table 2**), with ‘White Crystal’ highest and ‘Bicolor Fruit’ lowest (9.6). Central sectors (3–4) consistently showed peak values, declining toward the top (1) and base (5). Fructose content ranged 37.40–44.08 mg/g (**Figure 3**), peaking in central sectors for most cultivars, with ‘Jindu No.1’ highest overall. Glucose followed similar patterns (5.01–6.33 mg/g; **Figure 4**), with ‘Bicolor Fruit’ exhibiting the highest glucose content but ‘Bai Yulong’ the lowest. Significant spatial differences (*p* < 0.05) underscored genotypic specificity in sugar distribution. Notably, the central region emerged as a “sugar hotspot” in most varieties, hinting at varietal-specific metabolic regulation that could be linked to enzyme activities.

**Table 2:** Average soluble solids in different parts of pitaya fruits from four varieties.

Variety	Site 1	Site 2	Site 3	Site 4	Site 5	Average value
Jindu No.1	10.9	15.4	17.1	16.3	10.1	14.0
White Crystal	13.3	14.9	16.7	16.5	15.1	15.3
Bicolor Fruit	9.6	10.4	14.3	15.1	9.7	11.8
Bai Yulong	12.0	13.6	15.8	14.2	10.9	13.3

One-way ANOVA results: Cultivar effect: F_3,16_ = 1.82, *p* = 0.185; Sector effect: F_4,15_ = 5.66, *p =* 0.006.

### 3.3 Genotypic divergence in sucrose metabolism enzyme activities

**Figure 5:**
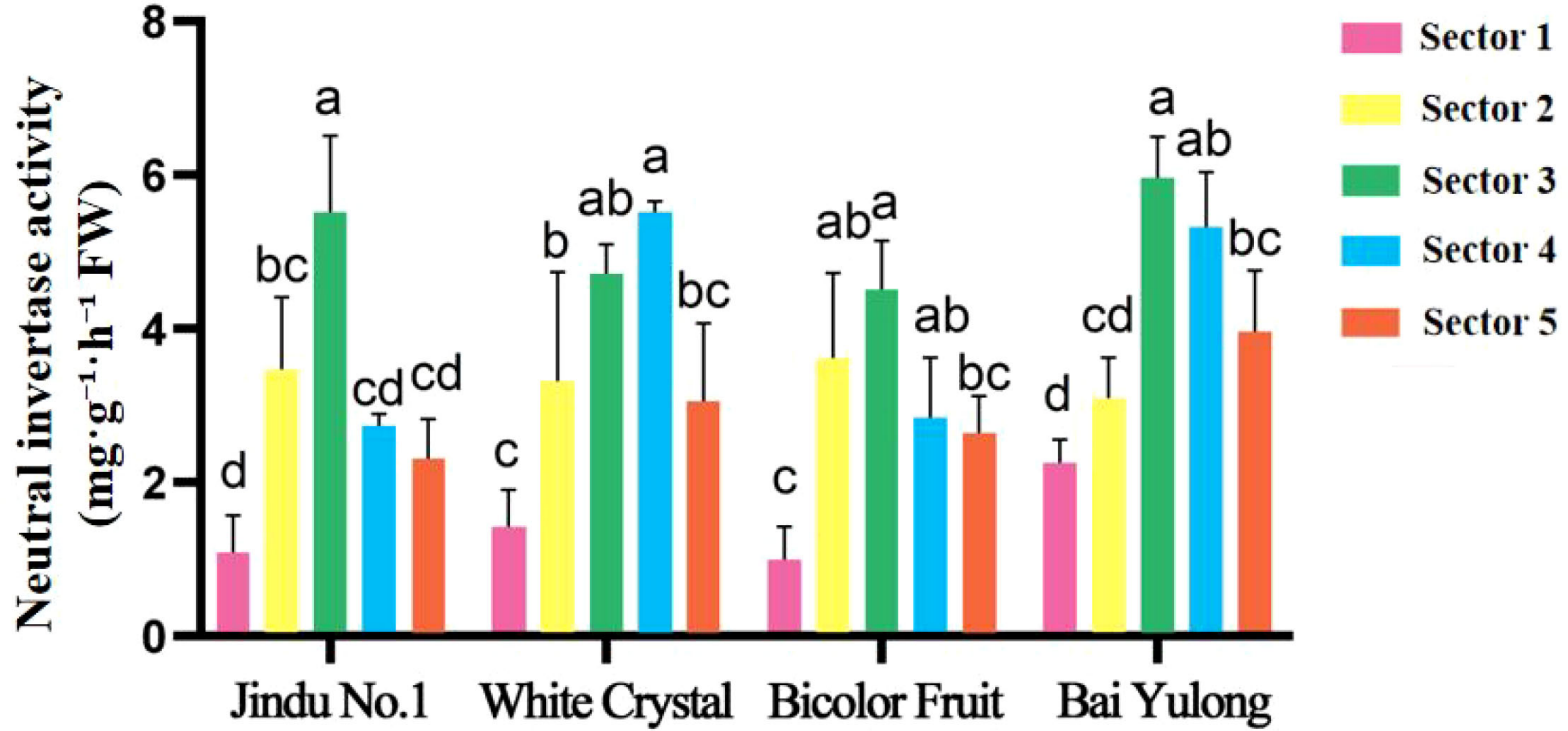
Neutral invertase activity in different parts of four varieties of pitaya. Data are means ± SD (n=3). For each cultivar, one-way ANOVA was performed with fruit sector as the factor. Different lowercase letters indicate significant differences among sectors within the same cultivar (Tukey’s HSD, *p* < 0.05).

**Figure 6:**
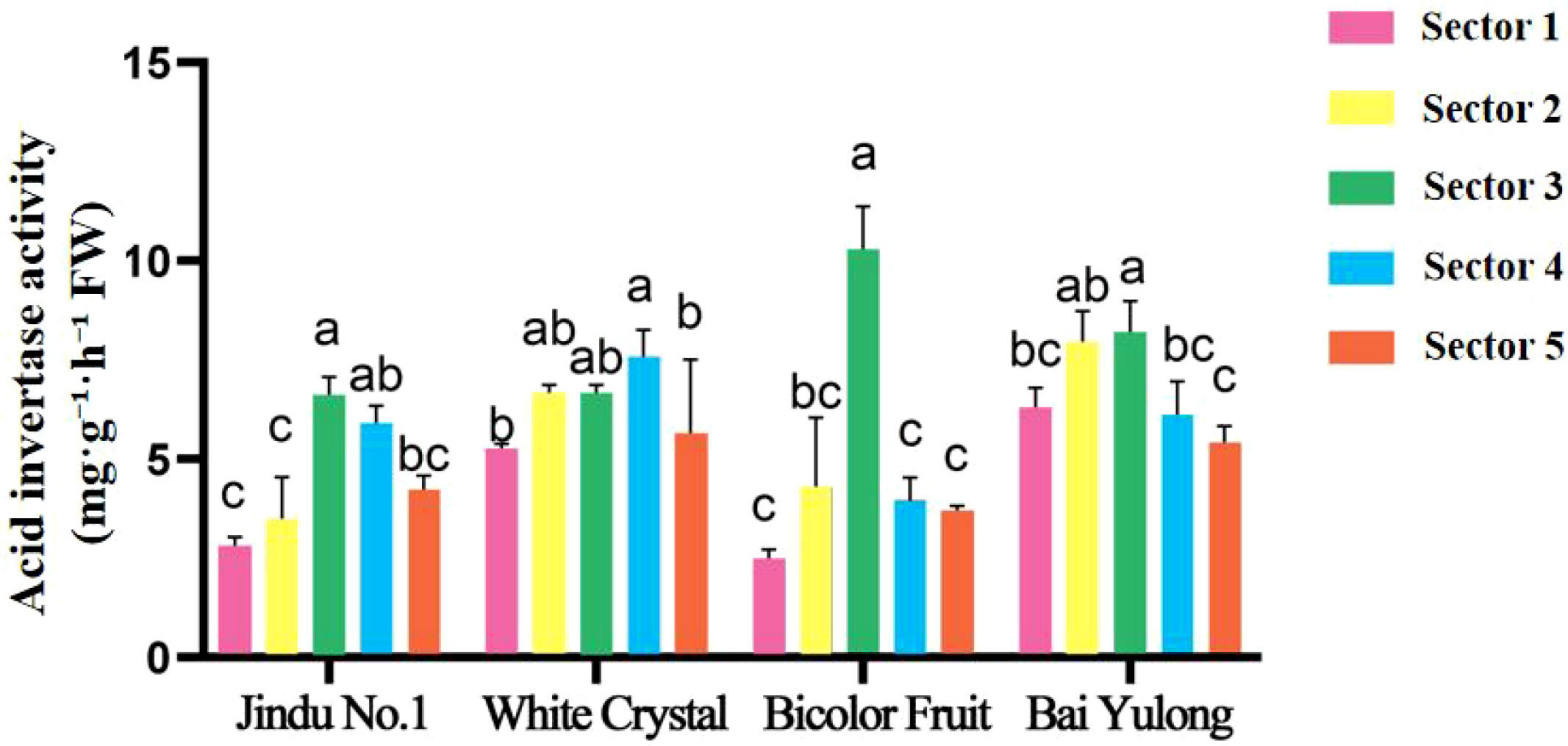
Acid invertase activity in different parts of four varieties of pitaya. Data are means ± SD (n=3). For each cultivar, one-way ANOVA was performed with fruit sector as the factor. Different lowercase letters indicate significant differences among sectors within the same cultivar (Tukey’s HSD, *p* < 0.05).

**Figure 7:**
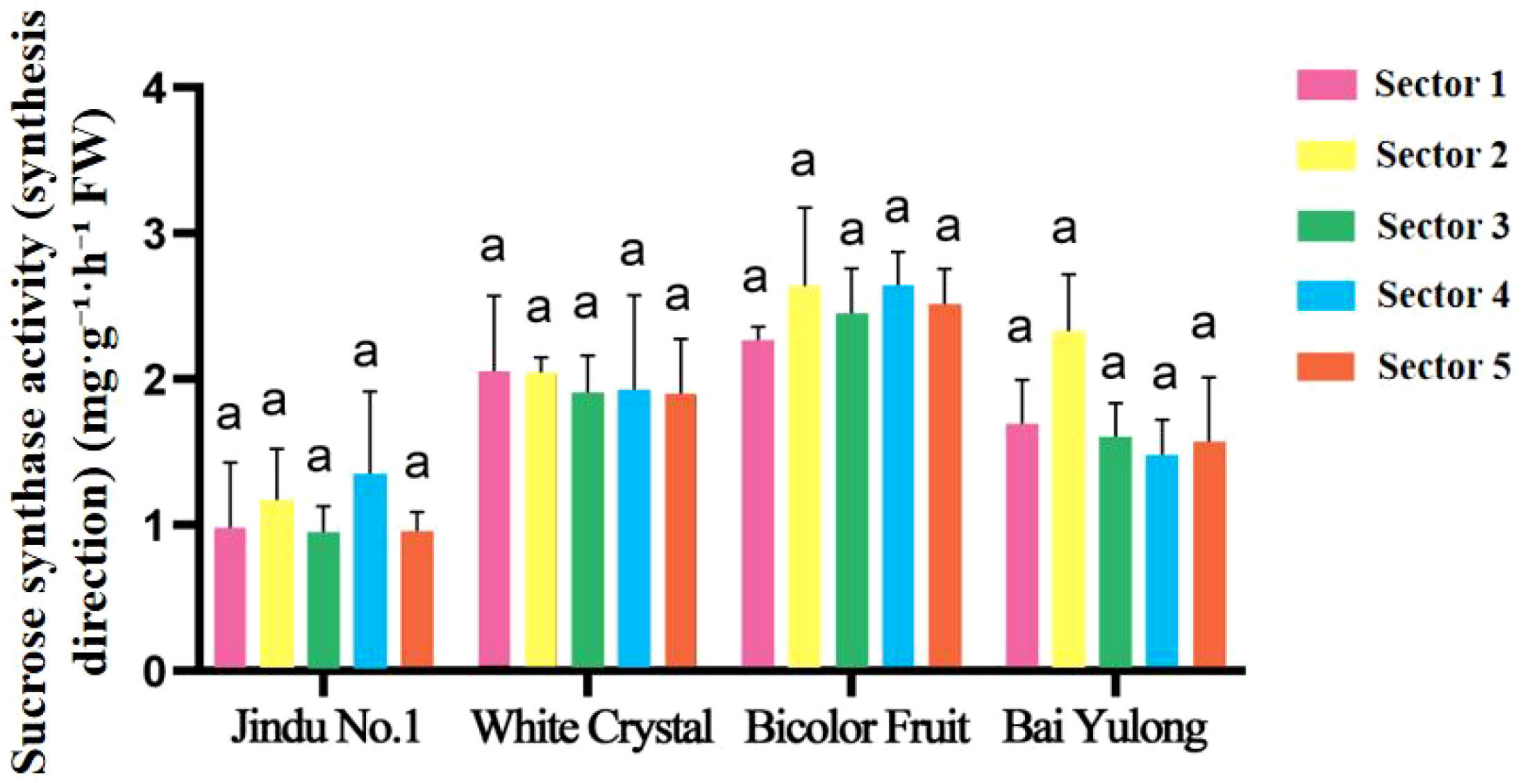
Sucrose synthase activity (synthesis direction) in different parts of four varieties of pitaya. Data are means ± SD (n=3). For each cultivar, one-way ANOVA was performed with fruit sector as the factor. Different lowercase letters indicate significant differences among sectors within the same cultivar (Tukey’s HSD, *p* < 0.05).

**Figure 8:**
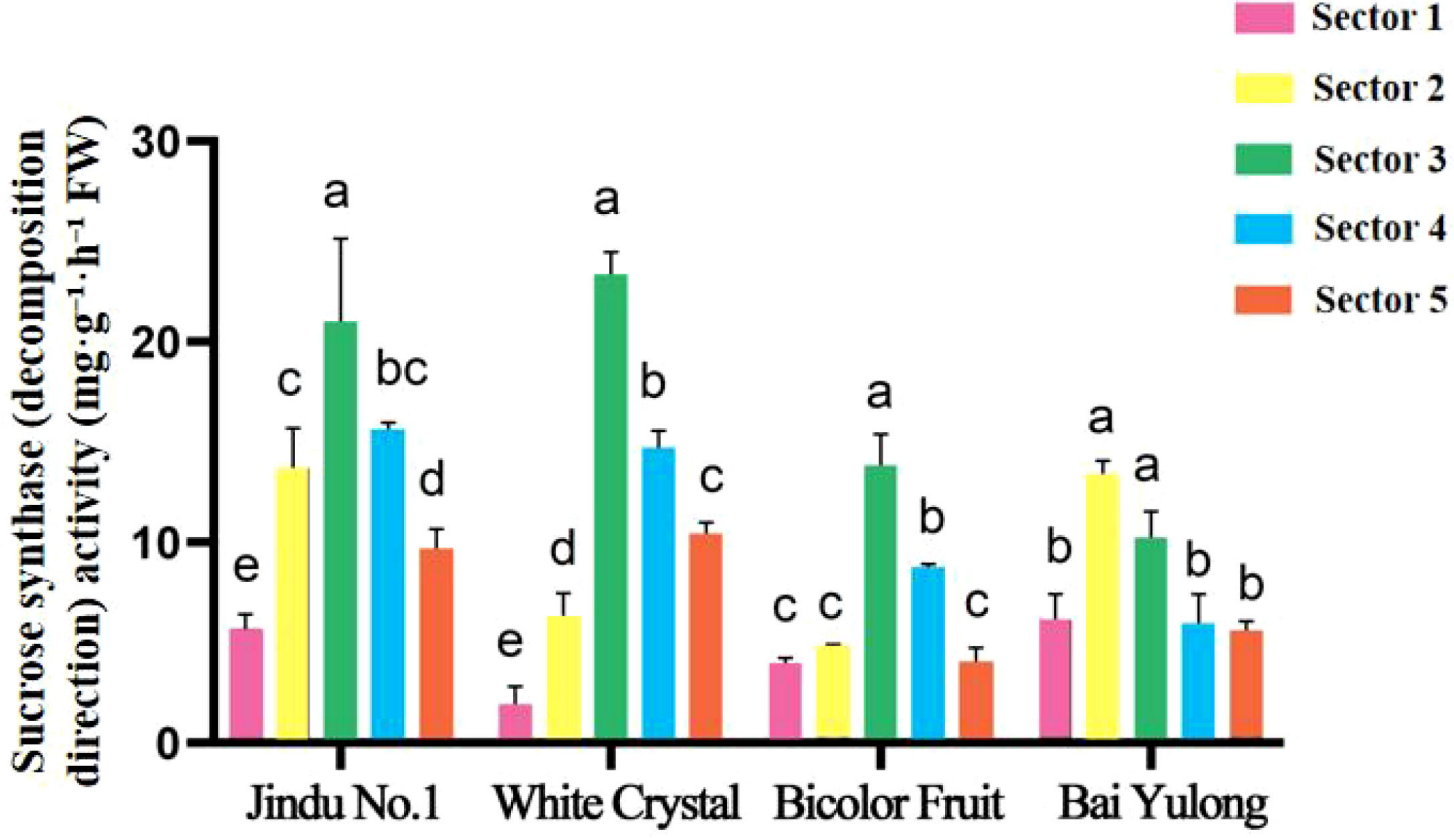
Sucrose synthase (decomposition direction) activity in different parts of four varieties of pitaya. Data are means ± SD (n=3). For each cultivar, one-way ANOVA was performed with fruit sector as the factor. Different lowercase letters indicate significant differences among sectors within the same cultivar (Tukey’s HSD, *p* < 0.05).

**Figure 9:**
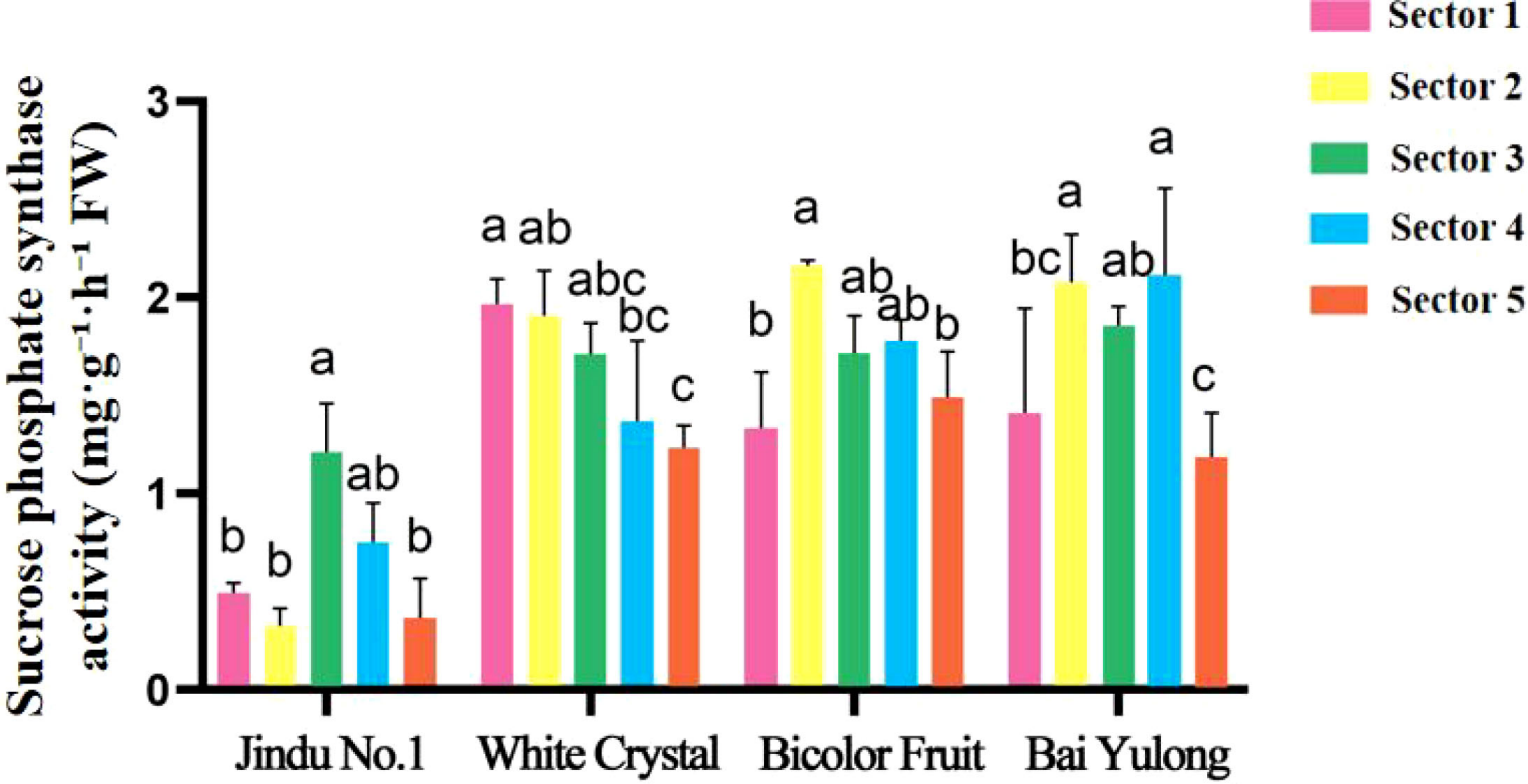
Sucrose phosphate synthase activity in different parts of four varieties of pitaya. Data are means ± SD (n=3). For each cultivar, one-way ANOVA was performed with fruit sector as the factor. Different lowercase letters indicate significant differences among sectors within the same cultivar (Tukey’s HSD, *p* < 0.05).

Enzyme activities exhibited striking genotypic and spatial variations. NI activity was highest in sector 3 of ‘Jindu No.1’ (**Figure 5**), with no significant differences in other cultivars. AI peaked in central sectors across varieties (**Figure 6**), particularly in ‘Jindu No.1’ and ‘Bicolor Fruit’. SS-synthesis activity appeared numerically higher in ‘Bicolor Fruit’ compared to other cultivars (**Figure 7**), with peak values exceeding 2.5 mg·g^−1^·h^−1^ in sectors 3–4, but showed no significant intra-cultivar variation (F_4,15_ = 1.12, *p* = 0.346). In contrast, SS-cleavage activity exhibited both inter-cultivar (F_3,16_ = 41.32, *p* < 0.001) and intra-cultivar variation (F_4,15_ = 4.076, *p* = 0.020), with ‘Jindu No.1’ showing the highest overall activity and significant peaks in central sectors (**Figure 8**). These results indicate functional divergence in SS directionality among genotypes. SPS varied irregularly (**Figure 9**), with no clear patterns. These results reveal functional divergence in SS—cleavage-dominant in ‘Jindu No.1’ versus synthesis-dominant in ‘Bicolor Fruit’—indicating a pivotal role in genotypic sugar metabolism.

### 3.4 Correlation and PCA: integrating traits for comprehensive evaluation

Pearson correlations showed strong positive links between fruit weight and size traits (*r* = 0.979–0.989, *p* < 0.01, indicated as ** in **Table 3**), but variable associations with sugars (**Table 3**). PCA extracted two components (cumulative variance 89.711%; **Tables 4**, **5**), with PC1 (51.97%) representing sugar metabolism (high loadings for soluble solids (0.881), edible rate (0.880), **Table 4**) and PC2 (37.75%) morphology (high positive loadings factors for single fruit weight (0.991) and transverse diameter (0.772); **Table 4**). Comprehensive scores ranked quality as ‘Bai Yulong’ (0.55) > ‘Jindu No.1’ (0.3) > ‘White Crystal’ (0.09) > ‘Bicolor Fruit’ (–0.94) (**Table 5**). The model illuminated trade-offs, such as ‘Jindu No.1’s morphological strength but moderate sugar efficiency, providing a framework for quality assessment.

**Table 3:** Correlation coefficients (r) between fruit quality traits of four varieties of pitaya.

Index	Single fruit weight/g	Peel weight/g	Longitudinal diameter/mm	Transverse diameter/mm	Thick- skinned/mm	Fruit shape index	Soluble solids
Single Fruit Weight/g	1.000						
Peel Weight/g	0.989**	1.000					
Longitudinal Diameter/mm	0.987**	0.988**	1.000				
Transverse Diameter/mm	0.979**	0.951**	0.974**	1.000			
Thick-skinned /mm	0.681	0.924	0.897	0.090	1.000		
Fruit Shape Index	0.547	0.788	0.952*	-0.182	0.859	1.000	
Soluble Solids	0.038	-0.190	-0.544	0.659	-0.328	-0.745	1.000

* indicates significant correlation (*p* < 0.05), ** indicates extremely significant correlation (*p* < 0.01).

**Table 4:** Component matrix.

Fruit quality indicators	Principal component 1	Principal component 2
Soluble Solids	0.881	0.156
Edible Rate	0.88	-0.31
Fruit Type Index	-0.873	0.475
Fructose	0.781	0.248
Single Fruit Weight	-0.073	0.991
Transverse Diameter	0.634	0.772
Longitudinal Diameter	-0.683	0.72
Glucose	-0.601	-0.719

**Table 5:** Main scores and comprehensive ranking of four pitaya fruits.

Variety	Score in each component		Comprehensive score	Ranking
	y1	y2	y	
Jindu No.1	-0.17	1.02	0.3	2
White Crystal	-0.03	0.28	0.09	3
Bicolor Fruit	-0.5	-1.79	-0.94	4
Bai Yulong	0.69	0.5	0.55	1

## 4 Discussion

This study provides a comprehensive comparative analysis of fruit quality and sucrose metabolism across four pitaya genotypes, revealing that genotypic variations in sugar accumulation are intricately linked to the differential regulation of key metabolic enzymes, particularly the bidirectional activity of sucrose synthase (SS). By integrating morphological traits, spatial sugar profiles, and enzyme activities, we not only quantified phenotypic diversity but also uncovered mechanistic insights into the “big-but-not-sweet” dilemma plaguing the pitaya industry. These findings extend beyond descriptive comparisons, offering a physiological framework for understanding how phenotypic differences among commercially grown cultivars manifest in quality traits, even in the absence of detailed pedigree information.

A central discovery is the functional divergence of SS, which appears to define an “ideal” metabolic mode for high-sugar accumulation in pitaya. In ‘Jindu No.1’ and ‘Bai Yulong’—the top-ranked cultivars—elevated SS-cleavage activity correlated strongly with higher hexose (glucose and fructose) levels, facilitating sucrose breakdown into readily utilizable sugars that enhance sweetness (**Table 6**). This aligns with the enzyme’s role as a reversible glycosyltransferase, where cleavage direction supports sink strength by providing uridine diphosphate (UDP)-glucose for glycolysis and hexose buildup (Stein and Granot, 2019). Conversely, ‘Bicolor Fruit’ exhibited dominant SS-synthesis activity (**Figure 7**), potentially channeling carbons toward structural polysaccharides or secondary metabolites like betalains, which are abundant in pigmented varieties (Ibrahim et al., 2018), thereby reducing soluble sugar pools. This trade-off mirrors patterns in other fruits; for instance, in citrus, high SS-cleavage promotes hexose accumulation in sink tissues, while synthesis direction favors starch or cell wall formation (Lowell et al., 1989). Similarly, in litchi, varietal differences in sugar composition are tied to SS balance, with cleavage-dominant modes yielding sweeter arils (Yang et al., 2013). Our correlation analyses further substantiate this: positive associations between hexoses and SS-cleavage (r = 0.406–0.929) across cultivars suggest that enhancing SS-cleavage could be a breeding target for “high-sugar” genotypes, potentially through genetic selection or CRISPR-based editing of SS isoforms (Ruan, 2014). However, this mode may come at a cost to fruit size, as seen in ‘Jindu No.1’s moderate morphology despite high sweetness, highlighting the need for balanced selection in polygenic traits.

**Table 6:** Correlation coefficients of soluble sugars and related enzyme activities in four pitaya varieties.

Variety	Sugar component	NI	AI	SS decomposition direction	SS synthesis direction	SPS
Jindu No.1	Fructose	0.936*	0.654	0.929*	0.182	0.641
	Glucose	0.705	0.328	0.649	0.039	0.576
White Crystal	Fructose	0.644	0.656	0.717	-0.192	0.204
	Glucose	0.424	0.579	0.406	0.214	0.534
Bicolor Fruit	Fructose	0.199	-0.219	0.042	0.848	0.634
	Glucose	0.722	0.779	0.623	0.040	0.538
Bai Yulong	Fructose	0.007	0.968**	0.895*	0.637	0.442
	Glucose	0.048	0.714	0.774	0.668	0.917*

* indicates significant correlation (*p* < 0.05), ** indicates extremely significant correlation (*p* < 0.01).

The observed spatial heterogeneity in sugar distribution and enzyme activities adds another layer of complexity, reflecting genotypic adaptations in resource allocation within the fruit. Central sectors (2–4) consistently served as “sugar hotspots,” with peak fructose and glucose contents (**Figures 3**, **4**) and elevated activities of AI, NI, and SS-cleavage (**Figures 5**–**8**), likely due to proximity to vascular bundles that enhance assimilate transport (Nomura et al., 2005). This gradient—declining toward the top and base—parallels findings in pineapple, where basal regions accumulate more sugars owing to stronger sink activity and enzyme localization (Zhang et al., 2012). In pitaya, genotypic variations amplified these patterns; for example, ‘Jindu No.1’ showed pronounced central peaks in SS-cleavage (**Figure 8**), suggesting enhanced metabolic flux in core tissues, possibly regulated by tissue-specific gene expression (Zhang et al., 2022). Such spatial dynamics underscore the concept of sink strength heterogeneity, where enzymes like AI and SS act as metabolic gates, modulating carbon partitioning in response to developmental cues (Sturm, 1999). Environmentally, factors like light and nutrients could exacerbate these gradients (Sosa et al., 2020), implying that cultivation practices might optimize uniformity in lower-ranked varieties like ‘Bicolor Fruit.’

Principal component analysis (PCA) provided a robust integrative tool, distilling multidimensional data into interpretable axes that reveal deeper quality trade-offs. PC1 (sugar metabolism, 51.966% variance) and PC2 (morphology, 37.745%) effectively ranked cultivars (**Table 5**), with ‘Bai Yulong’ excelling in both, embodying an equilibrated phenotype. This ranking illuminates evolutionary compromises under artificial selection. ‘Jindu No.1’—with high PC2 (morphology) but low PC1 (sugar metabolism)—exemplifies the ‘big-but-not-sweet’ phenotype, where breeding for size has inadvertently favored carbon allocation to structural growth over soluble sugar accumulation. This trade-off mirrors patterns observed in other domesticated fruits: in tomato, selection for larger fruit size has been linked to reduced sugar content due to both dilution and altered expression of sugar metabolism genes (Tieman et al., 2017). Conversely, ‘Bai Yulong’ achieves high scores in both components, suggesting that this genotype may have retained or rebalanced metabolic efficiency despite its large size. This ‘equilibrated’ phenotype is rare and valuable, indicating that artificial selection need not always sacrifice quality—a finding with implications for breeding programs aiming to overcome the size-sweetness trade-off. In contrast, ‘Bicolor Fruit’s negative scores in both components highlight inefficiencies in carbon utilization, potentially linked to its pigmentation demands (Li et al., 2018). Beyond ranking, PCA’s component loadings (**Table 4**) offer mechanistic insights: negative correlations between fruit shape index and soluble solids (r = –0.745; **Table 3**) suggest that elongated fruits may dilute sugar concentration due to increased volume, a hypothesis testable in broader germplasm (Pan et al., 2017). Notably, glucose exhibited negative loadings on both PC1 (−0.601) and PC2 (−0.719), indicating that glucose content contributed inversely to the principal components representing sugar metabolism and morphology. This suggests that glucose may not be the primary determinant of overall sweetness variation among pitaya genotypes, possibly due to its relatively stable concentration across cultivars or its role as a metabolic intermediate rapidly converted to other sugars or utilized in respiration. In contrast, fructose and soluble solids showed strong positive loadings on PC1, reinforcing their importance as key sweetness contributors. These findings align with the observation that SS-cleavage activity, which produces hexoses including glucose and fructose, is more closely associated with fructose accumulation and overall sugar profile, whereas glucose alone may not reflect the efficiency of sucrose breakdown or sink strength. Therefore, glucose should be interpreted cautiously in quality evaluation, and greater emphasis should be placed on fructose and enzyme activity traits for breeding high-sugar pitaya varieties. This model advances quality evaluation from univariate metrics to holistic frameworks, akin to PCA applications in apple breeding for flavor-metabolite integration (Aprea et al., 2017), and could guide genomic selection by prioritizing traits like SS activity in high-throughput screening.

Despite these advances, limitations warrant consideration. Our analyses were conducted at the physiological level, capturing enzyme activities but not underlying molecular regulators such as gene expression or post-translational modifications, which could vary diurnally or developmentally (Huber and Huber, 1996). Additionally, while cultivars were grown uniformly, subtle microenvironmental influences (e.g., soil heterogeneity) cannot be entirely ruled out, though our sampling minimized this. Future research should employ transcriptomics and proteomics to validate SS divergence (e.g., isoform-specific expression; Zhang et al., 2022) and explore agronomic interventions, such as nitrogen modulation, to enhance SS-cleavage without compromising yield (Perween and Hasan, 2019). Integrating genome-wide association studies (GWAS) with our PCA model could identify QTLs for “high-sugar” modes, accelerating molecular breeding.

## 5 Conclusion

In conclusion, this study identifies SS-cleavage dominance as a key metabolic mechanism distinguishing high-sugar pitaya cultivars (‘Bai Yulong’ and ‘Jindu No.1’) from low-sugar ones (‘Bicolor Fruit’). While genotypic variation in fruit traits is expected, our findings reveal that the directional balance of a single enzyme—sucrose synthase—explains a substantial portion of this variation and provides a physiological target for breeding. By elucidating spatial patterns, enzyme correlations, and PCA-derived trade-offs, we provide a mechanistic blueprint for overcoming the ‘big-but-not-sweet’ challenge that has arisen from artificial selection for yield. These physiological biomarkers and integrative evaluation tools offer practical value for precision breeding programs aimed at developing sweeter, market-competitive pitaya varieties, thereby contributing to the sustainable advancement of tropical fruit agriculture.

## Data Availability

The original contributions presented in the study are included in the article/supplementary material. Further inquiries can be directed to the corresponding authors.
